# The effect on HIV transmission and cost-effectiveness of programmes for female sex workers in East, Central, and Southern Africa: a modelling study

**DOI:** 10.1016/S2214-109X(24)00224-9

**Published:** 2024-09-01

**Authors:** Loveleen Bansi-Matharu, Paul Revill, Issac Taramusi, Richard Steen, Sungai T Chabata, Joanna Busza, Collin Mangenah, Sithembile Musemburi, Fortunate Machingura, Nicola Desmond, Primrose Matambanadzo, Maryam Shahmanesh, Raymond Yekeye, Owen Mugurungi, Frances M Cowan, James R Hargreaves, Andrew N Phillips

**Affiliations:** Institute for Global Health, University College London, London, UK; Centre for Health Economics, University of York, York, UK; National AIDS Council, Harare, Zimbabwe; Department of Public Health, Erasmus University, Rotterdam, Netherlands; Centre for Sexual Health and HIV AIDS Research (CESHHAR) Zimbabwe, Harare, Zimbabwe; Department of Population Health, London School of Hygiene & Tropical Medicine, London, UK; Centre for Sexual Health and HIV AIDS Research (CESHHAR) Zimbabwe, Harare, Zimbabwe, International Public Health Department, Liverpool School of Tropical Medicine, Liverpool, UK; Centre for Sexual Health and HIV AIDS Research (CESHHAR) Zimbabwe, Harare, Zimbabwe; Centre for Sexual Health and HIV AIDS Research (CESHHAR) Zimbabwe, Harare, Zimbabwe; International Public Health Department, Liverpool School of Tropical Medicine, Liverpool, UK, Malawi-Liverpool Wellcome Trust Clinical Research Programme, Liverpool School of Tropical Medicine, Blantyre, Malawi; Centre for Sexual Health and HIV AIDS Research (CESHHAR) Zimbabwe, Harare, Zimbabwe; Institute for Global Health, University College London, London, UK, Clinical Research Department, Africa Health Research Institute, Mtubatuba, South Africa; National AIDS Control Programme, Harare, Zimbabwe; AIDS and TB Directorate, Ministry of Health and Child Care, Harare, Zimbabwe; Centre for Sexual Health and HIV AIDS Research (CESHHAR) Zimbabwe, Harare, Zimbabwe, International Public Health Department, Liverpool School of Tropical Medicine, Liverpool, UK; Department of Population Health, London School of Hygiene & Tropical Medicine, London, UK; Institute for Global Health, University College London, London, UK

## Abstract

**Background:**

HIV prevalence and incidence has declined in East, Central, and Southern Africa (ECSA), but remains high among female sex workers (FSWs). Sex worker programmes have the potential to considerably increase access to HIV testing, prevention, and treatment. We aimed to quantify these improvements by modelling the potential effect of sex worker programmes at two different intensities on HIV incidence and key health outcomes, and assessed the programmes’ potential cost-effectiveness in order to help inform HIV policy decisions.

**Methods:**

Using a model previously used to review policy decisions in ECSA, we assumed a low-intensity sex worker programme had run from 2010 until 2023; this resulted in care disadvantages among FSWs being reduced, and also increased testing, condom use, and willingness to take pre-exposure prophylaxis (PrEP). After 2023, three policy options were considered: discontinuation, continuation, and a scale-up of the programme to high-intensity, which would have a broader reach, and higher influences on condom use, antiretroviral therapy (ART) adherence, testing, and PrEP use. Outputs of the key outcomes (the percentage of FSWs who were diagnosed with HIV, on ART, and virally suppressed; the percentage of FSWs with zero condomless partners, and HIV incidence) were compared in 2030. The maximum cost for a sex worker programme to be cost-effective was calculated over a 50-year time period and in the context of 10 million adults. The cost-effectiveness analysis was conducted from a health-care perspective; costs and disability-adjusted life-years were both discounted to present US$ values at 3% per annum.

**Findings:**

Compared with continuing a low-intensity sex worker programme until 2030, discontinuation of the programme was calculated to result in a lower percentage of FSWs diagnosed (median 88·75% *vs* 91·37%; median difference compared to continuation of a low-intensity programme [90% range] 2·03 [–4·49 to 10·98]), a lower percentage of those diagnosed currently taking ART (86·35% *vs* 88·89%; 2·38 [–3·69 to 13·42]), and a lower percentage of FSWs on ART with viral suppression (87·49% *vs* 88·96%; 1·17 [–6·81 to 11·53]). Discontinuation of a low-intensity programme also resulted in an increase in HIV incidence among FSWs from 5·06 per 100 person-years (100 p-y; 90% range 0·52 to 22·21) to 4·05 per 100 p-y (0·21 to 21·15). Conversely, comparing a high-intensity sex worker programme until 2030 with discontinuation of the programme resulted in a higher percentage of FSWs diagnosed (median 95·81% *vs* 88·75; median difference compared to discontinuation [90% range] 6·36 [0·60 to 18·63]), on ART (93·93 *vs* 86.35%; median difference 7·13 [–0·65 to 26·48]), and with viral suppression (93·21% *vs* 87·49; median difference 7·13 [–0·65 to 26·48]). A high-intensity programme also resulted in HIV incidence in FSWs declining to 2·23 per 100 p-y (0·00 to 14·44), from 5·06 per 100 p-y (0·52 to 22·21) if the programme was discontinued. In the context of 10 million adults over a 50-year time period and a cost-effectiveness threshold of US$500 per disability-adjusted life-year averted, $34 million per year can be spent for a high-intensity programme to be cost-effective.

**Interpretation:**

A sex worker programme, even with low-level interventions, has a positive effect on key outputs for FSWs. A high-intensity programme has a considerably higher effect; HIV incidence among FSW and in the general population can be substantially reduced, and should be considered for implementation by policy makers.

**Funding:**

Wellcome Trust.

## Introduction

HIV prevalence and incidence in the general population in East, Central, and Southern Africa (ECSA) have decreased considerably over the last 20 years but remain high among female sex workers (FSWs), with a prevalence of up to 68%,^1^ and an incidence up to nine times higher than in the general female population.^1–6^ Therefore, FSWs are in particular need of access to HIV diagnostic testing and primary prevention and treatment, to both improve their health and wellbeing and to reduce onward transmission.

The main factor driving these high prevalence and incidence levels in FSWs is most likely to be the high number of partners with whom this group have condomless sex, partially due to difficulties in negotiating condom use with clients, or clients’ offers to pay higher fees for condomless sex.^7^ Psychological, social, and economic factors such as violence, low education levels among FSWs, poor mental health, and substance use are also known to significantly affect HIV infection rates among FSWs. Furthermore, FSWs and other women who have transactional sex might be less able to use general HIV services than women not having transactional sex.^8,9^ Reasons for this include stigma and discrimination from health-care workers, and issues such as precarious housing situations, moving localities within a country, and competing needs, for example time commitments, transport costs, and the cost of medicines.^1,8^ These factors, alongside the widespread criminalisation of sex work in many countries in the region, means that providing HIV services to FSWs remains complex. Donor funding specifically for health services for sex workers in some ECSA countries does exist, but these services can be highly variable in terms of coverage, scale, and quality; in many settings, the needs of sex workers are not adequately addressed. This gap in funding was highlighted by UNAIDS in 2023; a 90% funding gap was reported for HIV prevention for key populations compared with the funding need for 2025.^10^

In a study published in 2006, it was estimated that between 0·4% and 4·3% of female people aged 15 to 49 years in Africa sell sex.^11^ More recent data suggest that the median number of FSW is around 1·5% of the adult female population.^12^ Of FSW living with HIV, estimates suggest up to two-thirds have a detectable viral load, although these estimates differ substantially between countries (lowest in Zimbabwe; 19%),^3,13,14^ and the proportion has declined rapidly over time with the roll-out of antiretroviral therapy.^3,5–7^ It has also been reported that over 50% of FSW self-report having condomless sex with a client in the last month,^3^ suggesting that the potential for onward transmission of HIV is substantial.

Health services specifically for FSW are scarce in the ECSA region, but where sex worker programmes are in place, their potential to have a considerable effect on services such as condom distribution, pre-exposure prophylaxis (PrEP), risk reduction counselling, HIV testing, linkage to care, and viral suppression has been shown.^15–17^ It is important to quantify the effect of such programmes in order to inform allocation of HIV prevention and care resources. This effect is not limited to the direct services a sex worker-specific programme might provide, but can also include supporting engagement in non-sex-worker specific services which FSWs can also use.

Using the HIV Synthesis model, which has been previously applied to review policy decisions in ECSA, we estimated the potential effect of sex worker programmes on HIV incidence and health outcomes, as well as assessing their potential cost-effectiveness, in order to inform future decisions about HIV prevention, care prioritisation, and resource allocation.

## Methods

### Overview

HIV Synthesis is an individual-based simulation model of HIV transmission, progression, and the effect of antiretroviral therapy (ART) that has been described previously.^18^ In brief, each time the model is run, a simulated population of adults is generated. Variables are updated every 3 months from 1989, and include age, sex, the number of short-term condomless sex partners, the presence of a long-term condomless sex partner, use of PrEP, HIV testing, and HIV acquisition. Additional variables for people with HIV include viral load, CD4 cell count, use of specific ART drugs, adherence to ART, resistance to specific drugs, and risk of HIV-related death. A 3-month period was used in the model to balance practicalities of model running computing time and capturing changing values of key variables.

### Modelling initiation of sex work and sexual behaviour

Setting scenarios are generated by sampling parameter values to represent a range of settings in ECSA and to incorporate uncertainty in model assumptions. Details of these assumptions can be found in the appendix (pp 93–116). 1000 setting scenarios were generated. Key model outputs in 2023 and corresponding observed data from countries across ECSA are shown in the appendix (pp 2–3). Observed estimates were generally within the 90% ranges from the model, suggesting model runs (setting scenarios) were indicative of countries across ECSA.

In this study we have used the terms FSWs and women interchangeably. As the underlying data sets included self-reporting, we assumed anyone self-identifying as female and a sex worker fell into the FSW category, without stratifying for transgender individuals.

For all women in the model, the lifetime susceptibility of initiating sex work (which might, for example, be dependent on economic hardship) is classed as low, medium, or high, reflecting different social circumstances. Among women aged 15–49 years with a medium or high susceptibility for initiating sex work the probability of initiating sex work in any 3-month period is dependent on four factors. First, whether the susceptibility is medium or high; second, age (older women have lower probabilities than younger women); third, the overall population levels of sexual risk behaviour; and fourth, whether the woman has previously been a sex worker. Once a woman has initiated sex work, the probability of stopping sex work is dependent on the base rate of stopping sex work, their age, and overall population levels of sexual risk behaviour. In any 3-month period in which a woman is considered to be an FSW, she might not be continually active as an FSW. Hence, she may have zero short-term condomless partners in any given 3-month period due to either being inactive in sex work, or using condoms consistently. Five sexual behaviour groups are defined for FSW to be in for a given 3-month period: first, no short-term condomless partners in the 3-month period; second, a low number (one to three) of short-term condomless partners (the exact number is determined by sampling from the values with equal probability); third, a medium number (four to 20) of condomless short-term partners; fourth, a high number (21–51) of condomless short-term partners; and fifth, a very high number (51–150) of short-term condomless partners. A woman can switch between groups with certain transition probabilities between 3-month periods. Further details can be found in the appendix (p 22).

We assume that, in the absence of a dedicated support programme for sex workers, FSWs have lower access to support and health services than other women.^19,20^ The extent of these disadvantages is difficult to estimate, and hence was sampled from a wide range of probabilities informed by expertise of the study team (appendix pp 108–09). In brief, we assumed higher rates of treatment interruption, higher rates of being lost at diagnosis, and reductions in ART adherence. We did not assume any disadvantages in terms of access to HIV testing, which was assumed to be the same for all women.

### Programme intensity levels and effects

We modelled a low-intensity sex worker programme from 2010 onwards. By intensity in this context, we are referring to the effect on condom use, ART adherence, HIV testing, and PrEP use in FSW who attend the programme. As with disadvantages sex workers might have, it is difficult to accurately estimate the effect of sex worker programmes, and effect will also vary across programmes in different settings. However, sex worker programmes have shown improved adherence and HIV viral suppression rates.^3,21^ We therefore assumed that the modelled sex worker programme could have varying effects on the overall disadvantages faced by FSWs, either reducing, negating, or even resulting in a health advantage for sex workers over other women (eg, ART counselling might result in sex workers being less likely to interrupt treatment; table 1). Sex worker programmes were also assumed to provide the following additional benefits: first, 6-monthly HIV testing in up to 50% of sex workers; second, the number of condomless partners reduced by two-thirds in up to 10% of sex workers (ie, greater condom use), willingness to take PrEP increased in up to 10% of sex workers, and chances of persistence of a sexually transmitted infection (STI) lasting beyond 3 months reduced by up to 20%. For the low-intensity programme, up to a 10% probability of entering the programme per 3 months was assumed, with assumed default of continued engagement thereafter, but with a probability of discontinuing care of up to 10% per 3-month period. Modelled outputs at baseline (2023) with a low-intensity programme in place since 2010 are shown in table 2. Across the range of setting scenarios, 1·57% (90% range 0·51–4·27) of women aged 15–49 years were FSWs with average duration of current sex work 4·79 years (2·45–7·47). Over a 3-month period, a median of 66·94% FSWs were predicted to have one or more condomless partners, suggesting a high risk of acquisition and transmission. Among FSWs, median values for HIV incidence and HIV prevalence were 6·37 per 100 person-years and 42·53%, respectively. 90·03% of FSW with HIV were diagnosed, 89·17% of those diagnosed FSWs were on ART, and 88·48% of those on ART were virally suppressed.

For high-intensity intervention programmes, we assumed a higher likelihood of being engaged with the programme than a low-intensity intervention programme: up to 30% probability per 3 months and a lower assumed probability of discontinuing care of up to 3% per 3-month period. Benefits of a high-intensity intervention programme were assumed to be considerably greater than those in a low-impact programme; these are shown in table 1.

### Cost estimates

Assuming a cost-effectiveness threshold of US$500 (and lower thresholds of $300 and $100 for sensitivity analyses), disability-adjusted life-years (DALYs) for the whole adult population were used to calculate the maximum cost of a programme for it to be deemed cost-effective. Country-specific thresholds are generally uncertain, and while higher thresholds have been recommended in, for example, South Africa, $500 per DALY averted is probably at the upper end in most lower-income settings in ECSA.^22^ The cost-effectiveness analysis was conducted from a health-care perspective; costs and health outcomes were both discounted to present US$ values at 3% per annum. This was estimated by calculating the net DALYs averted—ie, DALYs averted by the sex worker programme plus the DALYs that can also be averted with the cost savings offered.

### Outcomes

The key outcomes for comparison between the three modelling scenarios at the 2030 endpoint were the percentage of FSW diagnosed with HIV, the percentage on ART, the percentage virally suppressed, and the percentage of FSW with zero condomless partners. We also compared HIV incidence amongst FSW and all the above outputs for the general population. Cost efficacy of the three programme scenarios was calculated on the maximum total cost and cost per sex worker per year over a 50-year period. Incidence of HIV per 100 person-years (100 p-y) and HIV prevalence were key outcomes in accordance with the UNAIDS 90–90–90 targets.

### Role of the funding source

The funder of the study had no role in study design, data analysis, data interpretation, or writing of the report.

## Results

Outputs in 2030 for each of these scenarios are shown in table 3. Compared with continuing a low-intensity sex worker programme, discontinuation of the programme resulted in a lower percentage of FSWs diagnosed (median 88·75% *vs* 91·37%; median difference [90% uncertainty range] 2·03 [–4·49 to 10·98]), a lower percentage of those diagnosed currently taking ART (86·35% *vs* 88·89%; 2·38 [–3·69 to 13·42]), and a lower percentage of FSWs on ART with viral suppression (87·49% *vs* 88·96%; 1·17 [–6·81 to 11·53]). The percentage of FSWs with no condomless partners per 3 months was also lower (28·89% *vs* 33·25%; 3·03 [–0·49 to 14·18]). HIV incidence among sex workers was predicted to be higher if the low-intensity sex worker programme was discontinued (5·06 per 100 p-y *vs* 4·05 per 100 p-y, difference –0·73 [–6·26 to 3·14]). The 90–90–90 indicators, HIV incidence and HIV prevalence in the general population, were all very slightly improved if a sex worker programme continues at low intensity compared with discontinuation of the programme (table 3).

If a sex worker programme offered high-intensity interventions and at a higher coverage, a considerable improvement was seen in key outputs by 2030 (table 3). There was an increase in the percentage of FSWs diagnosed (88·75% *vs* 95·81%), the percentage of those diagnosed on ART (86·35% *vs* 93·93%), and the percentage of those on ART who are virally suppressed (87·49% *vs* 93·21%). The percentage of FSWs with no condomless partners increased to 45·46% (median difference compared to discontinuation 13·51 [90 uncertainty range 4·54 to 35·36]), although there was no substantial change in the proportion of FSWs on PrEP (26·30% *vs* 26·51%). With a high-intensity intervention sex worker programme, HIV incidence among FSWs decreased to 2·23 per 100 p-y from 5·06 per 100 p-y with discontinuation of the programme (median difference –2·39 [90% range –10·33 to 0·79]). The changes in modelled outputs for FSWs also translated to positive changes for the wider population. In particular, HIV incidence and prevalence were predicted to decrease to 0·15 per 100 p-y and 5·94%, respectively (table 3). Over longer time horizons, incidence in the general population was expected to fall further with a high-intensity sex worker programme in place: 0·12 per 100 p-y in 2073 in the general population compared with 0·20 per 100 p-y if the programme was discontinued (median difference –0·07 [90% range –0·32 to –0·00]).

Costs and DALYs over a 50-year time period in the context of 10 million adults are shown in the figure and table 4. Overall costs did not include the running of a sex worker programme, but did include the costs of additional tests and ART which might occur because of the programme. Mean costs over 50 years are lower per year if the low-intensity sex worker programme continues compared with discontinuation of the low-intensity programme. Costs are even lower per year with the continuation of a high-intensity sex worker programme. This is true for individual components of the overall costs, such as ART costs. As expected, the cost of testing in sex workers is higher with a high-intensity programme, given the increased probability of 6-month testing. However, this does not translate into higher testing costs overall, which remain similar for the three options. DALYs are highest when the low-intensity programme is discontinued, and lowest with a high-intensity programme continued over a 50-year period.

**Figure 1:**
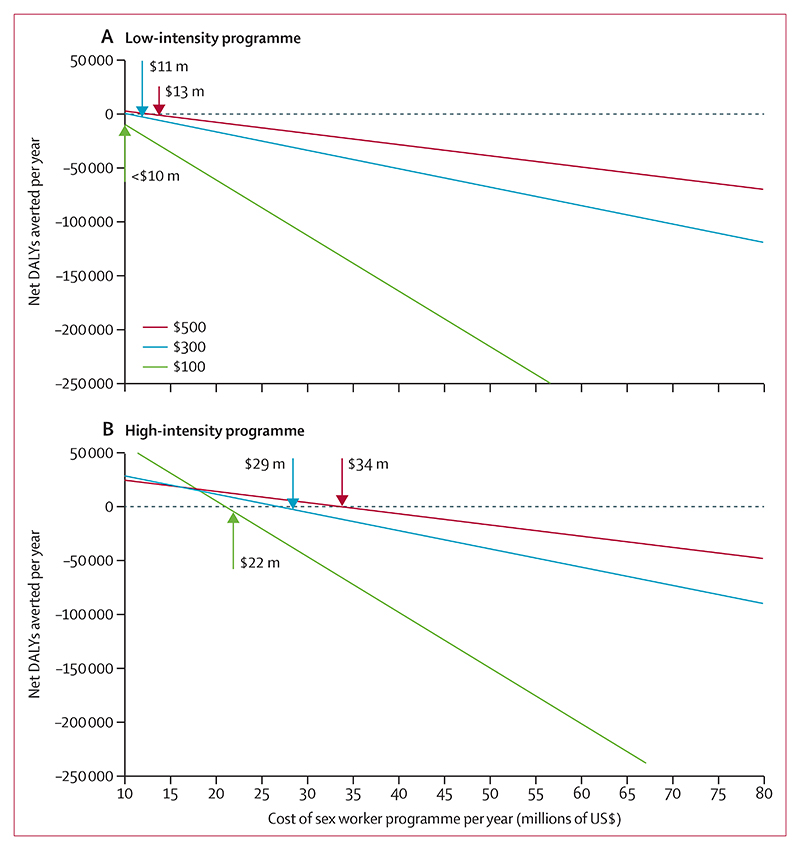
Net DALYs averted comparing a sex worker programme to discontinuation of the programme using cost-effectiveness thresholds of US$500, $300, and $100 Arrows show maximum cost of a sex worker programme for it to be cost-effective at the three thresholds. DALY=disability-adjusted life-year. m=million.

Taking these costs and DALYs into account for the 50-year period, along with a cost-effectiveness threshold of US$500, we calculated the net DALYs averted if a sex worker programme was in place using various costs of a sex worker programme (figure). A value of zero for the net DALYs averted signifies the maximum cost of a sex worker programme for it to be cost-effective. This maximum cost was US$13 million per year (90% range 0–50) on a low-intensity sex worker programme (approximately $172 per FSW in the population per year) and $34 million (0–105) per year on a high-intensity sex worker programme ($430 per FSW in the population per year; table 4). Using lower cost-effectiveness thresholds resulted in lower maximum costs of a sex worker programme for it to remain cost-effective. However, these costs were still substantial; using a threshold of $100, a maximum of $22 million per year could be spent on a high-intensity sex worker programme and still be cost-effective (figure 1). The net DALYs averted for any given spend on a sex worker programme would be expected to be even higher with a lower cost-effectiveness threshold, as the cost savings from the programme can be invested in high-intensity health interventions. Assuming a notional $150 cost per sex worker in the population, increased testing, higher condom use, increased STI services, and increased adherence were significantly associated with sex worker programmes being cost-effective (appendix p 10).

The maximum cost of a sex worker programme in order for it to remain cost-effective varied by HIV incidence in 2023 (table 5). In general, maximum costs were lower with lower incidence but in high-incidence settings (ie, where incidence was >0·5 per 100 p-y), maximum costs were considerably higher than the $13 million and $34 million calculated above ($21 million and $54 million, respectively).

## Discussion

Using the HIV Synthesis model we simulated 1000 setting scenarios representative of countries and regions across ECSA. We have shown that over the course of a fairly short period of time, from 2023 to 2030, our assumptions have resulted in even a sex worker programme with low-intensity interventions having a positive effect on prevention of HIV (increased condom use and uptake of PrEP), testing for HIV, and retention in care for FSWs, compared with discontinuing such a programme.

We have further shown that by increasing a low-intensity intervention programme to high-intensity interventions, manifested by, for example, a higher likelihood of 6-monthly testing, greater condom use, and ART counselling, FSWs are considerably less likely to have condomless sex (albeit only 45% with zero condomless partners, there is still a considerable reduction in HIV incidence), and more likely to be on PrEP, diagnosed, and virally suppressed over the course of the 7 years to 2030. While it is difficult to quantify the total effect of a sex worker programme, the assumptions made and results shown are in line with the HIV care cascade in existing FSW programmes.^15,21^

Importantly, as a result of a high-intensity intervention programme being in place, HIV incidence among FSWs is predicted to be lower in 2030 than the incidence predicted with a low-intensity intervention programme. Consequently, the incidence in the general population would also be substantively lower.

Our estimates are probably conservative, given the intervention was considered over a relatively short period of time. The maximum amount that can be spent on a sex worker programme for it to remain cost-effective is substantial: $34 million per year for a high-intensity programme in a setting of 10 million adults, of whom 1·6% of women aged 15–49 years are assumed to be FSWs, and the maximum cost is higher still in high-incidence settings. Even with lower cost-effectiveness thresholds, $22 million per year can be spent on high-intensity programmes and still remain cost-effective. Moreover, the net DALYs averted for any given spend on a sex worker programme would be expected to be higher if cost-effectiveness thresholds were lower. This is because the cost savings can be reinvested in high-effect health-care programmes addressing unmet health needs of the whole population. This implies that even the lowest resourced health-care and HIV programmes should be prioritising interventions aimed directly at sex workers, owing to the gains that will be made in other forms of health-care delivery as a result of cost saving.

We chose to assess differences in interventions in 2030, as this aligns with the Global AIDS Strategy 2021–2026 to end AIDS by 2030. HIV incidence at the population level is declining, and there is also evidence of such a decline among FSWs.^6^ This key population accounts for a small percentage of the wider population, usually less than 5%. However, HIV incidence among FSWs is still nine times higher than women in the general population in Africa,^6^ and FSWs have 30 times the risk of acquiring HIV compared with the general population globally.^23^ In West and Central Africa, data from 2021 showed nearly a quarter of all new HIV acquisitions were among FSWs (13% in East and Southern Africa).^23^ Up to 38% of all new infections in areas with high-to-moderate condom use could be attributable to sex work. This is significantly higher, up to 88%, in areas where there are no interventions in place for FSWs.^24^ Clients of FSW also play a key role in HIV transmission to their relationship partners, spreading HIV outside the FSW population, and hence addressing the prevention and treatment needs of FSWs has important benefits beyond the FSWs themselves.^25^

While ART provision is variable among sex worker programmes, those that do not provide ART can still improve linkage to care and counselling.^15–17^ Programmes can improve adherence and rates of viral suppression. In this modelling study there were considerable improvements from 2023 to 2030 in viral suppression rates among FSWs even with low-intensity intervention programmes in place compared with scenarios in which no sex worker programme was in place. Similar rates have been observed among FSWs in Zimbabwe,^3^ showing that our estimated rates are achievable. If a high-intensity programme was in place, viral suppression rates among FSWs were estimated to be close to those estimated for the general population.^21^ This has the potential for a considerable reduction in onward transmission.

While maintaining consistent condom use is often challenging in sex work, considerable evidence from Africa supports the importance of continuing strong condom programming and related STI services. Studies have shown increasing rates of self-reported condom use and declining or stabilising HIV incidence, prevalence, or both in the Democratic Republic of the Congo, Kenya, Zimbabwe, Senegal, Côte d’Ivoire, and Benin.^26–30^ Earlier modelling has estimated that increasing condom use and reducing curable STIs, especially in highly active sex work networks, act synergistically and independently from ART services to reduce HIV incidence and prevalence.^7^ In our analyses, we assumed sex worker programmes will result in a reduction in long-term STIs, provided that strong STI screening and treatment services are in place. We do also explicitly model an increased risk of HIV acquisition when another STI is present (appendix p 37), and thus the reduction in the proportion of people with STIs is also likely to have contributed to the lower incidence seen with a sex worker intervention programme. STI transmission in sex work has increased since ART became widely available.^31^ Inconsistent condom use due to behavioural disinhibition might well further increase if PrEP use increases as a result of sex worker programmes, making effective STI screening and treatment services for sex workers imperative.

While there is consensus that estimates for HIV incidence among FSWs are substantially higher than the incidence observed in the general population,^1–6^ HIV incidence estimates in FSWs vary considerably between studies. Our estimate of 6·37 per 100 p-y with an upper 90% centile of 31·95 per 100 p-y in 2023 was in the context of a low-intensity sex worker intervention programme, which is probably representative of existing sex worker programmes in the region. We have shown that considerable gains can be made towards lowering this incidence through more high-intensity sex worker intervention programmes, which increase the capability of sex workers to effectively use prevention and treatment by focusing on increased condom distribution, 6-monthly testing, and ART counselling. These gains could be even more effective by targeting those FSWs who are at highest risk.^21^ This could include, for example, peer-led microplanning tailored to individuals’ vulnerability and setting up self-help groups for FSWs to improve service delivery outcomes.

As in all modelling studies, there are limitations to our analyses. There are scant reliable data available for FSWs; data on sexual behaviour and condom use are based on self-reports and are prone to considerable bias. This uncertainty is reflected within our modelling by sampling key parameters, resulting in wide uncertainty ranges. We have also relied on self-reporting of sex, and thus used the terms women and female interchangably, although the model assumed sex to be assigned at birth. However, we feel our setting scenarios do reflect the range of epidemics seen both nationally and regionally in countries in ECSA. In particular, our baseline characteristics of FSWs were largely in line with FSWs seen under the Sisters programme in Zimbabwe which, as far as we are aware, is one of the most comprehensive sex worker programmes in the region.

With high ART coverage, prevention programmes, and counselling, HIV incidence in ECSA has declined significantly over the past 20 years. However, these declines are not evident to the same degree among FSWs, a population at high risk of transmission, and in which HIV incidence remains high. This is despite the existence of sex worker programmes of varying intensities. However, there is potential to make considerable change. Sex worker programmes provide essential support for FSW beyond the outputs we have analysed. They are key in connecting FSWs to mental health counselling, substance use treatment, gender-based violence screening, free legal services, and for general health and wellbeing; these are integral services in a marginalised community where depression, substance use, and violence can directly affect engagement in prevention and treatment programmes. By investing up to a maximum of $34 million annually in the context of 10 million adults in high-intensity sex worker programmes which focus on services such as condom distribution, counselling, and linkage to care, the programmes will remain cost-effective. HIV incidence among FSWs can be significantly reduced and viral suppression rates can be increased to be in line with those seen in the general population; this suggests that implementation of such programmes should be considered by policy makers.

## Supplementary Material

Supplementary Materials

## Figures and Tables

**Table 1:** Parameters relating to sex work programme effects sampled with equal likelihood according to sex worker programme intensity level for each setting scenario for female sex workers

	Low-level intensity* (per 3 months)	High-level intensity† (per 3 months)
**General parameters**		
Engagement with programme	1%, 3%, 5%, 10%	10%, 20%, 30%
Disengagement from programme	2%, 5%, 10%	1%, 3%
Greater condom use (condomless partners reduced by two-thirds)	5%, 10%	Up to 3-fold higher‡
6-monthly testing	20%, 35%, 50%	Up to 2-fold increase
Increase in willingness to take PrEP	5%, 10%	Up to 2·5-fold increase
Persistent STI reduction	10%, 20%	Up to 4-fold increase
**In relation to disadvantages**		
Interruption disadvantage reduced	30%, 50%, 70%	Up to 3-fold increase
Loss at diagnosis disadvantage reduced	30%, 50%, 70%	Up to 3-fold increase
Adherence disadvantage reduced	10%, 15%, 25%	Up to 4-fold increase

For a full list of studies reporting high and low level intensity, see appendix (pp 4–5). PrEP=pre-exposure prophylaxis. STI=sexually transmitted infection.

* Assumed since 2010.

† From 2023.

‡ A value for the low-level intensity is chosen at random and multiplied by the fold-increase to determine values for the high-level intervention. Parameters for the high-intensity programme were capped at the maximum possible effect.

**Table 2:** Modelled outputs on FSWs in 2023

	1000 runs, median (90% range)	Examples of studies showing influence of sex work programmes*
Percentage of FSW aged 15–49 years	1.57% (0·51–4·27)	Range across ECSA (0·3% to 2·8%; Malawi *vs* Burundi)
Average duration of current period of sex work, years	4·79 (2·45–7·47)	Zimbabwe (2015) 4·4 and (2021) 9·0; Kenya (2019) 8·7; Malawi (2018) 76% were sex workers for over 3 years; South Africa (2018) 8·0
Age of FSW, years		
15–19	10·69 (6·96–17·34)	Zimbabwe, aged 18–19 years (2015) 2% and (2021) 4%; Malawi (2018) 26% aged 13–19 years
20–24	25·58 (19·22–35·47)	Zimbabwe (2015) 13% and (2021) 17%; Ethiopia (2020) 52% aged under 25 years*; South Africa (2019) 9% to (2019) 13% aged 18–24 years; Kenya (2019) 21% aged under 25 years*; Rwanda (2019) 15% aged 18–24 years; Tanzania (2018) 44% aged 18–24 years; Malawi (2018) 19%
25–29	26·65 (20·94–32·57)	Zimbabwe (2021) 20% and (2015) 22%; Ethiopia (2020) 40% aged 25–34 years; South Africa (2019) 22% aged 25–29 years and 46% aged 25–34 years; Kenya (2019) 35% aged 25–34 years; Rwanda (2019) 50% aged 25–34 years; Tanzania (2018) 40% aged 25–34 years; Malawi (2018) 33% aged 25–34 years
30–39	32·35 (21·77–38·61)	Zimbabwe (2021) 36% and (2015) 41%; South Africa (2019) 24% aged 30–34 years, 19% aged 35–39 years, 36% aged 35–44 years; Kenya (2019) 44% aged over 35 years; Rwanda (2019) 36% aged over 35 years; Tanzania (2018) 16% aged 35–45 years; Malawi (2018) 32% aged over 35 years
Percentage of FSWs with no condomless partners (including inactive FSWs)	33·06% (9·42–57·27)	Zimbabwe (2021) 20% and (2015) 39%; Kenya (2019) 77% at last sexual encounterf; Rwanda (2019) 20% at last sexual encounterf; Tanzania (2018) 21% over the past 3 months†
Percentage of FSWs currently taking PrEP	16·09% (3·33–37·04)	Zimbabwe (2015) 17% on second visitf, (2021) 22% on second visitf but 0·4% when measured with urine samples; Rwanda (2019) 67% at a 6-month visit; Tanzania (2019) 8%
Percentage of FSWs with other STIs (apart from HIV)f	12·81% (7·21–19·80)	Ethiopia (2020) 16% with any history; Kenya (2019) 14%; Tanzania (2018) 6%; Malawi (2018) 30%
HIV among FSWs		
Percentage of FSWs with any resistance mutations	19·42% (1·49–52·59)	No data found
Incidence per 100 person-years	6·37 (0·12–31·95)	Range: 2·1 (Tanzania) to 7·1 (Zimbabwe, FSWs aged 15–24 years); South Africa: 4·6
Prevalence	42·53% (11·56–79·10)	Zimbabwe (2021) 46%; South Africa (2019) 62%; Togo (2017) 13%; Ethiopia (2020) pooled 18%, high 28%; Kenya (2019) 28%; Tanzania (2018) 8%; Malawi (2018) 53%
Percentage diagnosed	90·03% (75·88–94·58)	Zimbabwe (2021) 89%; South Africa (2019) 92%; Ethiopia (2020) 51%
Of those diagnosed, percentage on ART	89·17% (63·90–97·66)	Zimbabwe (2021) 95%; South Africa (2019) 87%; Ethiopia (2020) 93%
Of those on ART, percentage of viral load <1000 copies per mL	88·48% (62·02–99·59)	Zimbabwe (2021) 93%; South Africa (2019) 74%; Ethiopia (2020) 92%

All outputs are per 3-month period unless stated otherwise. For a full list of studies reporting programme effects, please see the appendix (pp 6–7). ART=antiretroviral therapy. ECSA=East, Central, and Southern Africa. FSW=female sex worker. PrEP=pre-exposure prophylaxis. STI=sexually transmitted infection.

* Includes 15–24 year age bracket.

† Observed data is generally based on self-reports which probably represent an underestimate of the true proportion of FSW with STIs.

**Table 3:** Effect of a sex work programme on key outputs in 2030

	Sex worker programme discontinued in 2023	Sex worker programme continues at low coverage and intensity from 2023	Sex worker programme at high coverage and intensity from 2023
**Population of FSWs**			
Percentage who visit the programme	0	42·91% (12·22 to 74·97)	78·20% (59·75 to 89·13)
Percentage tested in last year	57·67% (30·93 to 85·08)	63·67% (38·28 to 87·88)	79·13% (55·41 to 96·34)
Median difference compared to discontinuation	Ref	5·31 (–3·22 to 16·77)	18·95 (4·31 to 44·14)
Total percentage diagnosed	88·75% (74·04 to 95·51)	91·37% (77·44 to 97·21)	95·81% (86·92 to 99·94)
Median difference compared to discontinuation	Ref	2·03 (–4·49 to 10·98)	6·36 (0·60 to 18·63)
Of those diagnosed, percentage on ART	86·35% (54·39 to 96·46)	88·89% (62·31 to 97·47)	93·93% (78·36 to 98·42)
Median difference compared to discontinuation	Ref	2·38 (–3·69 to 13·42)	7·13 (–0·65 to 26·48)
Of those on ART, percentage with a viral load <1000 copies per mL	87·49% (62·04 to 97·98)	88·96% (66·52 to 98·20)	93·21% (79·35 to 100·0)
Median difference compared to discontinuation	Ref	1·17 (–6·81 to 11·53)	5·15 (–2·58 to 23·83)
Percentage of FSW with no condomless partners	28·89% (8·52 to 51·06)	33·25% (10·03 to 58·23)	45·46% (14·56 to 76·82)
Median difference compared to discontinuation	Ref	3·03 (–0·49 to 14·18)	13·51 (4·54 to 35·36)
Percentage of FSW on PrEP	26·30% (1·46 to 49·70)	27·81% (2·05 to 52·64)	26·51% (2·94 to 56·37)
Median difference compared to discontinuation	Ref	1·51 (–5·33 to 10·06)	2·51 (–10·40 to 18·43)
Percentage of FSW with long-term (>3 months) STIs	16·07% (9·96 to 21·60)	12·68% (7·40 to 18·94)	9·21% (4·01 to 14·39)
Median difference compared to discontinuation	Ref	–3·31 (–7·06 to 0·67)	–6·91 (–11·83 to –2·19)
Percentage of FSW with any resistance mutations	15·99% (0 to 48·63)	14·89% (0 to 46·42)	11·74% (0 to 40·91)
Median difference compared to discontinuation	Ref	–0·01 (–0·08 to 0·05)	–0·04 (–0·14 to 0·03)
HIV incidence per 100 person-years	5·06 (0·52 to 22·21)	4·05 (0·21 to 21·15)	2·23 (0·00 to 14·44)
Median difference compared to discontinuation	Ref	–0·73 (–6·26 to 3·14)	–2·39 (–10·33 to 0·79)
HIV prevalence	32·97% (7·26 to 70·04)	31·34% (6·92 to 68·65)	28·12% (5·73 to 65·70)
Median difference compared to discontinuation	Ref	–1·14 (–6·81 to 4·66)	–3·88 (–10·66 to 1·39)
**Entire population**			
HIV incidence per 100 person-years	0·21 (0.02 to 1·20)	0·19 (0·02 to 1·11)	0·15 (0·01 to 0·99)
Median difference compared to discontinuation	Ref	–0·02 (–0·17 to 0·08)	–0·05 (–0·30 to 0·04)
HIV prevalence	6·25% (1·11 to 23·95)	6·15% (1·11 to 23·73)	5·94% (1·04 to 23·30)
Median difference compared to discontinuation	Ref	–0·05 (–0·42 to 0·19)	–0·17 (–0·80 to 0·10)
Total proportion diagnosed	92·17% (86·61 to 96·36)	92·67% (87·23 to 96·67)	93·46% (88·61 to 97·08)
Median difference compared to discontinuation	Ref	0·36 (–1·01 to 2·31)	1·17 (–0·29 to 3·81)
Of those diagnosed, percentage on ART	96·19% (90·29 to 98·36)	96·30% (90·67 to 98·40)	96·49% (91·00 to 98·41)
Median difference compared to discontinuation	Ref	0·10 (–0·42 to 0·84)	0·27 (–0·26 to 1·35)
Of those on ART, percentage with a viral load <1000 copies per mL	93·91% (86·12 to 98·49)	93·97% (86·25 to 98·49)	94·14% (86·42 to 98·54)
Median difference compared to discontinuation	Ref	0·03 (–0·60 to 0·65)	0·13 (–0·44 to 0·92)

Differences are median (90% range). For a full list of studies reporting high and low level effects, please see the appendix (pp 8–9). ART=antiretroviral therapy. FSW=female sex worker. PrEP=pre-exposure prophylaxis. STI=sexually transmitted infection.

**Table 4:** Cost-effectiveness outputs to determine maximum cost of a sex worker programme for the programme to be cost-effective

	Sex worker programme discontinued in 2023	Sex worker programme continues at low intensity from 2023	Sex worker programme continues at high intensity from 2023
Overall costs*, millions of US$	140 (136 to 146)	137 (132 to 142)	132 (127 to 137)
Difference in overall costs compared to no programme	Ref	–3·8 (–4·2 to –3·3)	–9·2 (–9·8 to –8·6)
ART costs, millions of US$	42 (39 to 44)	40 (38 to 42)	38 (36 to 40)
Difference in ART costs	Ref	–1·4 (–1·6 to –1·3)	–3·5 (–3·7 to –3·2)
PrEP costs, millions of US$	6·3 (5·9 to 6·6)	6·0 (5·7 to 6·4)	5·6 (5·3 to 6·0)
Difference in PrEP costs	Ref	–0·2 (–0·3 to –0·2)	–0·6 (–0·7 to – 0·5)
Testing costs among FSWs, millions of US$	0·4 (0·3 to 0·4)	0·5 (0·4 to 0·5)	0·6 (0·6 to 0·6)
Difference in testing costs amongst FSWs	Ref	0·1 (0·1 to 0·1)	0·3 (0·2 to 0·3)
Testing costs overall, millions of US$	12 (12 to 12)	12 (11 to 12)	11 (11 to 12)
Difference in overall testing costs	Ref	–0·1 (–0·2 to –0·1)	–0·3 (–0·4 to –0·2)
Difference in DALYs compared to no programme	Ref	–7366 (–8124 to –6607)	–18 301 (–19 665 to –16 937)
Total costs			
Total spent on a sex worker programmef to remain cost- effective compared with no programmed, millions of US$ per year, mean (95% CI [90% range])	Ref	13 (12 to 14 [0 to 50])	34 (31 to 36 [0 to 105])
Total cost per sex worker per year§ in US$, mean (95% CI [90% range])	Ref	172 (152 to 192 [0 to 590])	430 (404 to 456 [0 to 1109])

Data are mean across runs (95% CI) unless otherwise stated. Costs and DALYs are given per year, over a 50-year time horizon from 2023. All costs and DALYs are discounted at 3% per year, over a 50 year time horizon in the context of 10 million adults. ART=antiretroviral therapy. DALY=disability-adjusted life-year. FSW=female sex worker. PrEP=pre-exposure prophylaxis.

* Excluding cost of the sex worker programme, but including the costs of any extra tests, extra people on ART, and extra PrEP users as a result of the programme.

† This does not include the costs of HIV testing, PrEP, ART, etc, for sex workers, only the costs of running the programme, and accounts for discounting per year.

‡ Maximum cost for which net DALYs are averted (see figure).

§ Calculated by dividing the maximum cost of a sex worker programme by the number of sex workers in 2023.

**Table 5:** Maximum cost of a sex worker programme for it to remain cost-effective, stratified by whole population incidence* in 2023

	Maximum cost per year for a low intensity sex worker programme to remain cost- effective†	Maximum cost per year for a high intensity sex worker programme to remain cost-effective†
**0–0·10 per 100 p-y**		
Cost in millions of US$, mean (95% CI [90% range])	3 (2·5 [0·20])	7 (5·9 [0·30])
Cost per sex worker in US$, mean (95% CI [90% range])	66 (42·90 [0·32])	140 (103·177 [0·644])
**0·11–0·30 per 100 p-y**		
Cost in millions of US$, mean (95% CI [90% range])	9 (7·10 [0·35])	22 (20·25 [0·60])
Cost per sex worker in US$, mean (95% CI [90% range])	131 (108·154 [0·529])	349 (311·388 [0·950])
**0·31–0·50 per 100 p-y**		
Cost in millions of US$, mean (95% CI [90% range])	12 (9·14 [0·45])	37 (34·41 [0·90])
Cost per sex worker in US$, mean (95% CI [90% range])	168 (132·204 [0·561])	522 (468·577 [0·1190])
**0·51–1·00 per 100 p-y**		
Cost in millions of US$, mean (95% CI [90% range])	21 (18·24 [0·60])	54 (48·60 [0·130])
Cost per sex worker in US$, mean (95% CI [90% range])	242 (201·284 [0·794])	597 (541·653 [0·1341])
**>1·00 per 100 p-y**		
Cost in millions of US$, mean (95% CI [90% range])	27 (21·32 [0·90])	62 (51·72 [0·180])
Cost per sex worker in US$, mean (95% CI [90% range])	314 (207·420 [0·894])	637 (544·730 [0·1857])

Costs and costs per sex worker are based on whole population incidence of HIV per 100 p-y. 100 p-y=100 person-years. DALY=disability adjusted life year.

‡ Whole population incidence of HIV was the strongest predictor of scenarios averting DALYs. See appendix (p 11) for other factors.

† Maximum cost for which net DALYs are averted.

## Data Availability

This is a modelling study and hence no new primary data were collected for this study. The model details have been reviewed, and are provided in the appendix.

## Research in context

### Evidence before this study

Female sex workers (FSWs) are a key population disproportionally affected by HIV. In East, Central, and Southern Africa (ECSA) the median number of FSWs is around 1·5% of the adult female population. Of FSWs living with HIV, estimates suggest up to two-thirds have an unsuppressed viral load. Rates of condomless sex are high, suggesting that the potential for onward transmission of HIV is substantial. Where programmes specifically for sex workers exist, there has been considerable effect on HIV prevention and care for FSWs. We searched Web of Science in English on June 30, 2023, using the terms “HIV”, “sex-worker” AND a list of countries in ECSA, AND “program” or “programme” to identify all articles (without restriction on design) reporting on sex worker programme outputs in ECSA. We found that the potential outcomes and cost-effectiveness of sex worker programmes had not been previously quantified. Filling this gap in the literature is vital to inform effective allocation of resources.

### Added value of this study

Using a well-established individual-based HIV transmission model, which has been previously used to contribute to key policy decisions, we assumed a low-intensity sex worker programme established since 2010, and considered three policy options going forward to 2030: continuation of the programme, discontinuation of the programme, and a scale-up of the programme to a high intensity. Key outputs were compared in 2030 and the maximum cost for a sex worker programme to be cost-effective was calculated. Our modelled results showed that even low-intensity programmes had advantages in terms of the HIV care cascade for FSWs. Compared with discontinuation of the programme, continuation of the programme resulted in higher proportions of FSWs diagnosed with HIV, on antiretroviral therapy, virally suppressed, and having zero condomless partners. A considerably higher improvement on these outcomes was seen with a high-intensity programme, which, when based on a population of 10 million adults, was deemed to be cost-effective if it cost a maximum of US$34 million per year.

### Implications of all the available evidence

Sex worker programmes of various levels exist in ECSA. By scaling and intensifying these programmes, there is potential to make considerable positive change. By investing up to a maximum of $34 million on FSW-specific services focusing on condom distribution, counselling, and linkage to care, HIV incidence among FSW can be significantly reduced, and hence such programmes should be considered for implementation by policy makers.
